# Remote South American Snakebite with Extensive Myonecrosis

**DOI:** 10.5811/cpcem.2016.11.31220

**Published:** 2017-01-24

**Authors:** Russel Means, Jannella Cabrera, Xavier Moreno, Richard Amini

**Affiliations:** * University of Arizona, Department of Emergency Medicine, Tucson, Arizona; † University of Cuenca, Cuenca, Ecuador; ‡ Macas General Hospital, Department of Emergency Medicine, Macas, Ecuador

## Abstract

This report describes a patient envenomated by a *Bothrops atrox*, common fer-de-lance viper, in the remote rainforest of eastern Ecuador and without access to definitive care for seven days. Antivenom was not administered; by the time of presentation to a hospital, he had suffered myonecrosis of his lower leg, which was treated with debridement and eventual skin graft. The ramifications of this long evacuation demonstrate the need for more accessible health services and educational outreach.

## INTRODUCTION

The estimated 400,000 snakebites that occur annually worldwide are a serious problem for the regions they affect, primarily in nations with lower income and resources.^1^ Over 1,000 snakebites occur each year in Ecuador, with the vast majority inflicted by various pit viper species of the *Bothrops* genus.^2^ Furthermore, *B. asper* is responsible for the majority of ophidic morbidity and mortality throughout Latin America. Studies have demonstrated that incidence and death rates (10 and 0.05 per 100,000 persons per year, respectively) in Ecuador are consistent with neighboring rural Brazil and Colombia.^3^,^4^ In a study of 187 bites treated with antivenom in the Amazon region of Ecuador, two cases resulted in death; common complications included cellulitis (16%), renal failure (8%), abscess (6%), compartment syndrome (6%), and local necrosis (5%).^2^ Multiple studies show that polyvalent antivenoms from a variety of southern Central or South America countries have similar efficacies at reducing death as well as the hemotoxic and myotoxic effects of vipers from other regions.^1^,^3^,^5^ This has allowed countries such as Costa Rica to produce the vast majority of antivenoms used in Latin America, so that other countries need not invest considerable resources into production. Despite this, antivenoms are often unavailable in remote and rural regions due to the need for refrigeration and restrictive cost ($300 U.S. per vial). This unfortunately results in delayed treatment and increased complications.^6^

### Patient Presentation

A 36-year-old Ecuadorian male was transferred from a rural health center to a district-level hospital with worsening right lower leg pain, edema, and skin discoloration following a snakebite in a remote area of the Amazon seven days prior. He stated that he was walking in the jungle barefoot when he sustained an unprovoked bite on his right ankle. He beheaded the snake and walked to a neighboring shaman who provided local herbs and prayer for one day. He required several days of remote travel by foot and canoe to arrive at a community where air evacuation could be arranged for the remainder of the 150 miles to the district hospital. He identified the snake as an “equis,” the common name in the area for the *B. atrox*. His associated symptoms included gingival bleeding, hematemesis, abdominal pain, oliguria, and shortness of breath. He denied fever, chest pain, or neurological changes.

Triage vital signs were temperature 36.7 °C, blood pressure 100/60 mm Hg, heart rate 104 beats/min, respiratory rate 20 breaths/min, and SpO2 95%. Physical exam revealed the patient in no acute distress. His lower leg was edematous with areas of hyperpigmentation and ulceration extending to the dorsal foot. Labs showed a 12-minute whole blood clotting time (WBCT, normal 80–160 seconds), creatinine of 3.25 (0.5–1.2 mg/dL), urea of 174 (7 to 20 mg/dL), and a normocytic anemia with a hemoglobin of 5.3 (13.5–17.5 g/dL) and hematocrit of 14.1(43–52%). Ultrasound of the leg showed preserved blood flow and a fluctuant (4 cm × 1 cm) area in the anterior compartment; incision and drainage yielded 4mL of purulent fluid. Abdominal ultrasound revealed decreased renal corticomedullary differentiation suggestive of inflammation without obstruction.

**Image 1:**
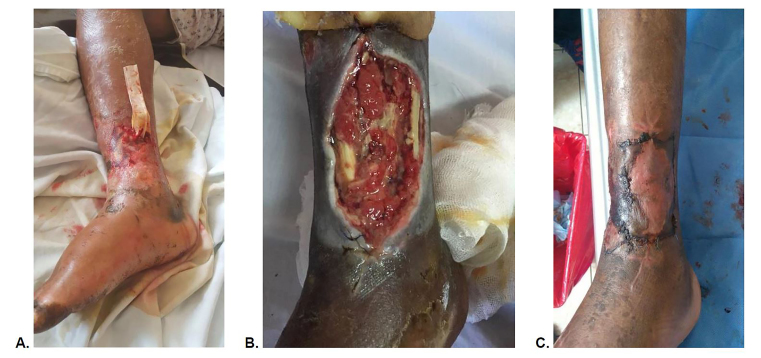
Progressive treatment of extensive myonecrosis in snakebite victim. **A.** 10 days after bite with drain insert into abscess. **B.** Two weeks after bite and extensive debridement. **C.** Status post graft hospital day 50.

The patient was diagnosed with a viper bite complicated by an abscess of the anterior tibial compartment and grade 3 renal insufficiency. The patient received two units of packed RBCs and was started on intravenous fluids, erythropoietin, iron, folic acid, enalapril, oxacillin, and acetaminophen. Subsequently, the patient was transitioned to clindamycin and started on enoxaparin for venous thrombosis prophylaxis. His necrotic tissue required staged debridements in the operating room, resulting in a large wound with exposed tendons; the wound was treated with daily bandage changes and sugar application for one month prior to successful skin grafting (Image 1). All care was provided by surgeons at the district level hospital in Macas with the exception of skin grafting in a tertiary care facility in Cuenca. We advised the patient to remain until full healing of the skin graft to reduce chance of infection in the remote jungle, but the patient wanted to return home to family and left against medical advice after two months of care.

## DISCUSSION

This case emphasizes the sequela of delayed definitive care in snakebites, which occurs far too commonly in rural areas of Ecuador. The risk of dying or developing serious complications from a snakebite is significantly higher (relative risk = 2.5) in those who do not receive antivenom in the first two hours after a snakebite.^5^ A polyvalent antivenom from Costa Rica was available for this patient once he reached the district-level hospital; however, given that the bite had occurred over one week prior to presentation, it was not administered. WBCT of greater than 20 minutes is often used to assess presence of viper bite; in this case WBCT of 12 minutes likely indicated decreased venom load and resolving coagulopathy. A study of the kinetics of *Bothrops* venom in rats showed an elimination half-life of 12 hours, with no circulating venom detected after seven days postenvenomation.^7^ It is unlikely that this patient would have experienced any benefit from antivenom if there was no remaining venom to be neutralized. Furthermore, few data exist on antivenom treatment timelines, but the high rate of adverse reactions to antivenoms (15–25%) makes discretionary use prudent.^1^

**Image 2:**
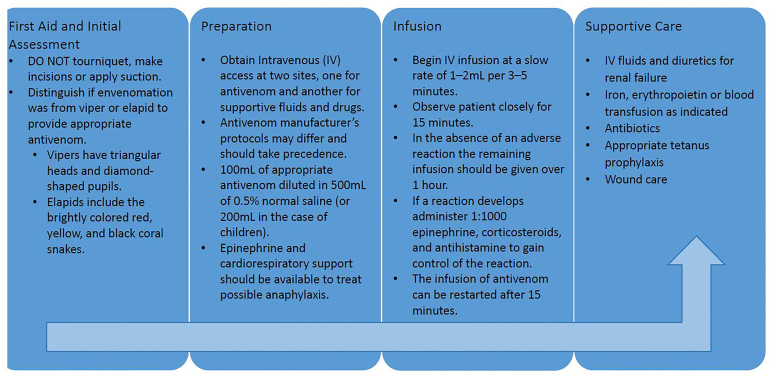
Protocol for snakebite treatment. Adapted from Howes *et al.*, 2005^2^ and De La Hoz *et al.*, 2008.^8^

Snakebite treatment protocol exists at the hospital in which the man was treated; however, given that the bite occurred over seven days prior to presentation, deviation from the protocol occurred and the patient was treated according to protocol and expert opinion. As a result, the patient was treated primarily using the indicated supportive treatment. Image 2 depicts the standard snakebite protocol used in Latin America that was developed in Costa Rica.^2^,^8^

Hemotoxic effects such as this patient’s gingival bleeding and anemia are common and mediated by a variety of venom components including zinc-dependent metaloproteinases.^1^,^3^,^9^ The extensive myonecrosis seen in this patient is thought to be largely due to toxic analogues of phospholipase A2 that result in phospholipid hydrolysis and compromise of the cell membrane. These toxic enzymes are the major target of the antibodies in antivenom, which neutralize and aid in their elimination.^1^,^2^,^3^ Regrettably, necrosis following viper envenomation frequently results in deficient skeletal muscle regeneration secondary to loss of basement membrane and axons. This patient had minimal capability to dorsiflex and invert his foot when he left the hospital two months after his ophidian accident.^3^

Our patient was geographically isolated and spent days receiving shamanistic care. These are common barriers for those living in remote rainforest communities that prevent prompt definitive care of snakebites. Approximately half of those bitten in Latin American countries will be treated first with traditional methods such as plants, chemical products, physical methods, or prayer.^5^ Unfortunately, these patients are isolated by long distances and geographical obstacles; traditional medicine is accessible and trusted, but only serves to delay antivenom administration. Efforts to educate traditional medicine practitioners and encourage rapid evacuation may serve to improve outcomes in remote regions. Despite Costa Rica’s high incidence of snakebite (14 per 100,000 per year) the mortality rate is lower than in most similar nations (0.02 per 100,000 persons per year).^4^ For 45 years the Instituto Clodomiro Picado in Costa Rica has had extensive educational outreach programs involving medical students and house staff to targeted high-risk communities using both Spanish and indigenous languages to improve knowledge of snakebite prevention and emergent treatment.^3^ Existing diet, sexual, and maternal health educational outreach programs to rural Latin America may benefit from pilot programs that bring awareness to snakebite preventative measures and the ramifications of delayed antivenom treatment. The morbidity and medical cost of our patient’s delay in care is a strong argument to provide more accessible healthcare to remote regions.
